# Outcomes of prolonged mechanic ventilation: a discrimination model based on longitudinal health insurance and death certificate data

**DOI:** 10.1186/1472-6963-12-100

**Published:** 2012-04-25

**Authors:** Hsin-Ming Lu, Likwang Chen, Jung-Der Wang, Mei-Chuan Hung, Ming-Shian Lin, Yuan-Horng Yan, Cheng-Ren Chen, Po-Sheng Fan, Lynn Chu Huang, Ken N Kuo

**Affiliations:** 1 Institute of Population Health Sciences, National Health Research Institutes, 35 Keyan Road, Zhunan, Taiwan; 2 Institute of Public Health, School of Medicine, National Yang-Ming University, No.155, Sec.2, Linong Street, Taipei, Taiwan; 3 Institute of Occupational Medicine and Industrial Hygiene, College of Public Health, National Taiwan University, No 17, Xuzhou Road, Taipei, Taiwan; 4 Department of Public Health, National Cheng Kung University College of Medicine, No.1, University Road, Tainan, Taiwan; 5 Departments of Internal Medicine and Environmental and Occupational Medicine, National Cheng Kung University Hospital, No.138, Sheng Li Road, Tainan, Taiwan; 6 Department of Internal Medicine, Chia-Yi Christian Hospital, 539 Jhongsiao Road, Chiayi, Taiwan

## Abstract

**Background:**

This study investigated prognosis among patients under prolonged mechanical ventilation (PMV) through exploring the following issues: (1) post-PMV survival rates, (2) factors associated with survival after PMV, and (3) the number of days alive free of hospital stays requiring mechanical ventilation (MV) care after PMV.

**Methods:**

This is a retrospective cohort study based on secondary analysis of prospectively collected data in the national health insurance system and governmental data on death registry in Taiwan. It used data for a nationally representative sample of 25,482 patients becoming under PMV (> = 21 days) during 1998-2003. We calculated survival rates for the 4 years after PMV, and adopted logistic regression to construct prediction models for 3-month, 6-month, 1-year, and 2-year survival, with data of 1998-2002 for model estimation and the 2003 data for examination of model performance. We estimated the number of days alive free of hospital stays requiring MV care in the immediate 4-year period after PMV, and contrasted patients who had low survival probability with all PMV patients.

**Results:**

Among these patients, the 3-month survival rate was 51.4%, and the 1-year survival rate was 31.9%. Common health conditions with significant associations with poor survival included neoplasm, acute and unspecific renal failure, chronic renal failure, non-alcoholic liver disease, shock and septicaemia (odd ratio < 0.7, *p *< 0.05). During a 4-year follow-up period for patients of year 2003, the mean number of days free of hospital stays requiring MV was 66.0 in those with a predicted 6-month survival rate < 10%, and 111.3 in those with a predicted 2-year survival rate < 10%. In contrast, the mean number of days was 256.9 in the whole sample of patients in 2003.

**Conclusions:**

Neoplasm, acute and unspecific renal failure, shock, chronic renal failure, septicemia, and non-alcoholic liver disease are significantly associated with lower survival among PMV patients. Patients with anticipated death in a near future tend to spend most of the rest of their life staying in hospital using MV services. This calls for further research into assessing PMV care need among patients at different prognosis stages of diseases listed above.

## Background

The healthcare arrangements for patients with prolonged mechanical ventilation (PMV), which is usually defined as mechanical ventilation (MV) for at least 6 hours per day for a period of 21 days or more [1], have been an issue in many countries with advanced medical technology. The United States began establishing respiratory intensive care units in the 1960s [2], while some European countries introduced facilities specifically for respiratory care in the 1980s [3].

The healthcare arrangements for such patients have also been a pressing issue in Taiwan, where a single-payer national health insurance (NHI) system started in 1995 and has been praised for its reasonable premiums and co-payments, and relatively short wait times for care [4,5]. The NHI provided care to all ventilator-dependent patients in intensive care units (ICUs) initially. Within three years, however, this care pattern led to a severe shortage of ICU capacity [6]. In 2000, the Bureau of National Health Insurance (BNHI) introduced "respiratory care centres" and "respiratory care wards" in hospitals to provide MV services to PMV patients 17 years of age or older in non-ICU settings.

As this program greatly increased the availability of PMV services, the number of PMV patients increased rapidly after launch of the program. The BNHI reported that the total number of patients receiving PMV in each of 2004 and 2005 reached 30,000 (0.13% of the Taiwan population), and the total annual expenses approached 27 billion New Taiwan dollars (NT$), which was around 6% of the total annual NHI budget. One BNHI report also reveals that the per-capita annual NHI expenses for patients receiving PMV in 2009 were 33 times that for all enrolees, and equal to 56 times the per-capita annual premium (NT$13,188 in 2009).

Controversial issues have emerged regarding the cost-effectiveness of extensive use of MV services, as well as distributive justice in healthcare allocation, because expenditures on this program crowded out resources for other healthcare in NHI. Another problem following the implementation of this program was that more physicians have to confront dilemmas when they have to respond to the preference of PMV patients or their families for withdrawing life-sustaining treatment after a period of PMV, as there have been much more PMV patients. It calls for more research on prognosis among PMV patients, because participants involved in decisions concerning terminal withdrawal of MV (including patients' families and physicians, and members in ethics committees for reviewing requests to withdraw PMV services) certainly need more reference materials in the process of decision-making to help them make better-informed decisions.

This study is an effort to generate helpful reference information in this regard through using a large national representative database to explore the following issues: (1) survival rates after a PMV incidence, (2) factors associated with survival after PMV, and (3) the number of days alive free of hospital stays requiring MV care after PMV.

## Methods

### Study design

This is a retrospective cohort study based on secondary analysis of prospectively collected data in the NHI system and governmental data on death registry. It is part of a research project on PMV care. The National Health Research Institutes (NHRI), the BNHI, and the Department of Health (DOH) approved the research project, and granted our request to construct a database linking NHI data and death-certificate data for patients receiving PMV care in the NHI. All individual identification numbers were scrambled by the BNHI for privacy protection.

### Setting

The study setting was in the NHI system. The NHI covers and protects almost all Taiwanese, and provides medical services in almost all outpatient visits and hospital admissions [7]. The NHI care program for PMV patients 17 years of age or older covers invasive ventilators, negative pressure ventilators, and positive pressure ventilators, under the condition that at least some use of an invasive ventilator or a negative pressure ventilator should be made prior to the first day of using a positive pressure ventilator [8]. Thus, PMV patients in the NHI only include those under MV care. Patients with a tracheostomy tube in situ not connected to a ventilator are not regarded as ventilator-dependent in the NHI.

While the BNHI defines PMV as ventilation care use for at least 21 consecutive days, a patient might have ventilation care use for fewer than 21 days at the time of entering the non-ICU care program for PMV in NHI, because the BNHI includes periods of discontinuation of MV care that had a length equal to or shorter than 4 days when aggregating a patient's time (in days) of use for judging the patient's eligibility of entering the program [8]. The BNHI sets limits on the lengths of stays in ICUs (an acute stage, < 21 days) and respiratory care centres (a subacute stage for weaning training, up to 42 days), but no limit for stays in respiratory care wards (a chronic stage or long-term care for those with little chance for weaning). Therefore, the NHI actually sets no limit on how long a patient may use MV care. The PMV program also includes homecare services for patients with a stable condition who are cared by family members or other caregivers at home. However, extremely few patients use homecare services, because the family have to pay much higher own expenses under this care pattern.

### Data sources

Two Taiwan governmental organizations provided original data for the study. The BNHI provided NHI data, and the Ministry of the Interior provided data from the national mandatory death registry system through the DOH. The BNHI maintains a comprehensive database of claims and registration data. The NHI database has detailed information on health services, procedures and prescriptions provided in the NHI, and their payments and times of use. It also includes data on diagnoses for patients, as well as background information on patients, physicians and healthcare institutions. The NHI system codes diagnoses using the International Classification of Diseases, Ninth Revision (ICD-9). The quality of NHI data is generally reliable, because the BNHI has been routinely auditing data submitted by healthcare institutions to prevent fraud in the NHI [9]. In Taiwan, it is also widely believed that death-certificate data are highly reliable, as death registry is mandatory in a well-maintained household registration system of Taiwan.

The database we acquired contains person-level longitudinal NHI claims and registration data for a national representative sample of patients who ever used invasive or non-invasive respiratory care in NHI during 1996-2007. This cohort included 2,619,534 patients, representing 10% of all NHI enrolees and 29% of all patients ever using any kind of respiratory care under NHI in these 12 years. This number of individuals was the maximal number of enrolees the government sets for application of NHI data use in 2008 (the year of our data application). We also acquired longitudinal NHI registration data for healthcare institutions and physicians.

### Establishment of a PMV patient cohort

We used SAS software version 9.1.3 (SAS Institute Inc., Cary, NC) to extract, organize, and link each patient's data on NHI registration and hospital care, and death certificate data. As the BNHI counts the lengths of short periods of discontinuation of MV care that was 4-day long or shorter when determining PMV status, we combined inpatient data for two admissions requiring MV for analysis if the readmission was within 4 days after the discharge of the previous admission. We defined such two admissions as a same episode of hospital stay for later analysis.

Although the BNHI has data on the exact dates of days with MV, the database we acquired only includes information on the amount of MV services for each medical order that was measured in days. Thus, we had to determine PMV status by counting the total number of days with MV in a hospital stay. For an episode of hospital stay that included two or more admissions requiring MV, we counted the number of days of short periods between two admissions when calculating the total number of days with MV. A hospital stay that had 21 or more days with MV was defined as one with a PMV incidence. To determine the time of a PMV onset, we assumed that MV services were offered right in the middle of a hospital stay with 21 or more days of MV to determine the 21^st ^day of MV use, which was the time of the PMV onset.

Taking into account the requirements of the NHI care program for PMV patients, we added two more criteria for selecting study patients: (1) use of invasive ventilators or negative pressure ventilators at the initiating stage of care, and (2) > = 17 years of age on the 21^st ^day of MV. We also confined the patient cohort to those becoming under PMV in or after 1998, when use of MV started to receive much attention. Finally, we decided to include in our project database only data for patients whose 1-year survival could be observed. Note that some patients used mechanical machines on and off, and had multiple episodes of PMV. Our study focuses on episodes that had at least a period of time of 365 days from all prior hospital stays with MV. This is based on an idea that findings from investigating outcomes among "new" patients with PMV can provide more reference information to physicians and policy makers, compared with results from examining future outcomes among patients who have been using MV services continuously or intermittently for months or even years. We finally identified 50,481 new patients who became under PMV in 1998-2006.

### Validation of data on PMV status and the time of PMV incidence

Because we counted the number of days of short periods between two admissions when calculating the total number of days with MV, some patients in our PMV patient cohort might actually order fewer than 21 days of MV services. To examine whether this might have a significant influence on accuracy of identification of PMV status, we investigated the proportion of patients in the study cohort who ordered MV services for fewer than 21 days. The proportion was 0.5%, suggesting that bias due to wrong identification of PMV status was minor. Furthermore, we calculated the ratio of "the total number of days with medical orders for MV services" to "the counted number of days with MV" (including short periods between two admissions requiring MV) for each patient. The average ratio of the 50,481 patients was 0.99. Only 2% of the patients had a ratio smaller than 0.8, while 87% had a ratio equal to 1. These results indicate that this project has minor inaccuracy in identifying PMV patients and determining the time of PMV onset.

### Study participants

We selected new PMV patients in 1998-2003 to assure a complete 4-year follow-up observation for each patient surviving the end of 2007. The inclusion criteria for these patients were: (1) continuous use of invasive ventilators, negative pressure ventilators, and/or positive pressure ventilators for at least 21 days, (2) use of invasive ventilators or negative pressure ventilators at the initiating stage of care; (3) > = 17 years of age on the 21st day of MV; (4) the date of the 21^st ^day of MV falling in 1998-2003; and (5) no use of invasive ventilators, negative pressure ventilators, and positive pressure ventilators for at least one year before the first day of this PMV event. We excluded patients with missing data for gender in descriptive analysis, and further excluded those with missing data for other explanatory variables in modelling survival prediction. The proportion of patients with missing data in the NHI database was very small. Exclusion of these patients from the study sample would not result in substantial bias.

### Variables

For each patient passing away before the end of 2007, we calculated post-PMV survival time as the period between the day of PMV incidence and the death date. For each patient who was alive at the end of 2007, we calculated post-PMV survival time that was censored at the end of 2007, and created a marker variable to denote right-censored data. Outcome variables for modelling survival prediction included binary variables showing 3-month, 6-month, 1-year, 2-year, 3-year and 4-year survival status after the PMV onset, with a value of 1 indicating successful survival and a value of 0 denoting failure. Data on factors associated with survival were from the NHI database. We counted the total number of days alive free of hospital stays with MV in the 4 years following a PMV incidence by subtracting the sum of the lengths of all hospital stays requiring MV in the 4 years following the PMV onset from the length of survival time in the immediate 4-year period after PMV.

### Modelling survival prediction

We estimated 3-month, 6-month, 1-year, and 2-year survival models to determine factors associated with survival and construct prediction functions for survival among PMV patients. For each survival model (3-month, 6-month, 1-year, or 2-year), we established a set of hospital and patient factors that were potentially influencing on survival. Other factors included diseases being diagnosed during the index hospital stay and during hospital stays in the immediate 1-year period before PMV.

Variables of hospital characteristics at the PMV onset included a set of binary variables indicating a hospital's accreditation level and region. The accreditation level of a hospital in Taiwan reflects the hospital's size and clinical capabilities [9]. The region of a hospital in Taiwan might capture effects on hospital behaviours or outcomes of the managerial pattern of a regional NHI office, as there are 6 regional NHI branch offices. We also included a continuous variable showing the year of PMV incidence. This variable was a proxy for capturing the effect of generous NHI coverage on PMV care on the trend in expanding MV care use to prolong a PMV patient's life over time after the new care policy.

Patient characteristics included a patient's gender and age, which are generally associated with disease prognosis. Also included was a set of binary variables indicating the urbanization level of a patient's NHI registration location that was measured according to population density and the local industrial pattern [10]. To estimate the effect of a patient's socioeconomic status, we included a set of binary variables showing the salary tertile.

Variables of diseases included a set of binary variables indicating conditions reported at the PMV onset, and a set of count variables showing the numbers of hospital admissions for treating various conditions in the immediate 1-year period before PMV. To classify diseases, we first used the Clinical Classifications Software developed by the U.S. Agency for Healthcare Research and Quality to categorize all diseases [11], and further reduced the number of disease classes down to 43 after discussion with physicians in related fields (see Additional file 1). While NHI claims data for reimbursement purposes do not provide very detailed information on disease categorization for each admission, the general quality of NHI data on disease diagnosis is acceptable [9]. After discussion with physicians in related fields, we believed that inpatient NHI data on disease diagnosis were adequate for this study, which looks into major and broad disease categories. We excluded variables for "respiratory failure" from the two sets of variables of diseases, because most physicians would include this diagnosis for PMV patients.

### Statistical analysis

We used Stata software version 9 (StataCorp, College Station, TX) for both descriptive statistics and multivariable regression analysis. Our descriptive analysis investigated survival up to 4 years, and examined the total length of time free of hospital stays with MV in the immediate 4-year period after PMV. Binary variables indicating successful survival were reported as percentage. The number of days alive free of hospital stays with MV was shown by selected percentiles (including minimum, median, maximum, and some others), mean and standard deviation.

Our estimation of prediction models focused on survival of 2 years or less, as a primary purpose of the prediction was to identify patients who were expected to die in a near future. Prediction of the likelihood of surviving a specific period of time can potentially yield information for facilitating communication with patients approaching the end of life and their family. We adopted logistic regression to identify factors associated with 3-month, 6-month, 1-year, and 2-year survival, and generate coefficient estimates for predicting a specific patient's probabilities of surviving different lengths of time. Compared to other contemporary methods of survival analysis, logistic regression is a very understandable method for generating predictions of survival likelihood and conducting subsequent comparison of predicted outcomes with actual outcomes to assess the quality of predictions.

Among explanatory variables for survival prediction, binary variables of hospital and patient characteristics were reported as percentage. Both the binary variables and the count variables indicating morbid conditions were reported as percentage on the basis of the proportion of patients with a non-zero value. To examine collinearity between explanatory variables, we calculated the variance inflation factor (VIF) for each variable, and the mean value of all VIFs. Results of the collinearity diagnostics indicate that inclusion of these variables would not result in damaging collinearity for multivariable regression analysis.

For comparison purposes, we used the probit regression method to estimate survival models. To investigate whether morbid conditions before PMV significantly influenced post-PMV survival, we also estimated survival models that excluded from explanatory factors the count variables showing the numbers of hospital admissions for treating various conditions in the immediate 1-year period before PMV. Additionally, we used a stepwise approach for selecting explanatory variables of diseases. We decided not to use the stepwise approach to select hospital and patient characteristics in model estimation, and included all of them. Because each hospital or patient feature was represented by a set of binary variables, it was thus not appropriate to use the stepwise approach for selecting variables among all these variables. We presented the results of survival models by reporting adjusted odds ratios (ORs) of explanatory variables and their 95% confidence intervals, and using a significant level of 5%. Data of 1998-2002 were used to estimate prediction functions; the 2003 data were employed to investigate model performance.

### The cutoff value of predicted survival probability for classifying patients

We selected 10% as the cutoff value of predicted probability to identify patients with low survival likelihood and classify patients after prediction. With this level of cutoff value, patients with a predicted probability of survival < 10% would be classified as a group that would die before the end of observation period, and those with a predicted probability of survival > = 10% would be classified as a group that would survive the whole observation period. There is no universal standard for choosing a cutoff value for prediction modelling; one criterion for judging appropriateness of the level of cutoff value is weighing benefits and harms due to decisions based on wrong predicted outcomes [12]. After discussion with some physicians in intensive care, we decided that 10% was an appropriately low survival probability for identifying patients who would be expected to pass away in a near future, to assure a small chance of wrongly categorizing a patient into a group with predicted death.

Using data for PMV patients of 2003, we compared predicted outcomes with actual outcomes, and further calculated four measures for reflecting the quality of predictions in different perspectives. The four measures and their formulae are below:

(1) sensitivity = (the number of patients with a predicted probability of survival > = 10%)/(the number of patients who actually survive the whole observation period);

(2) specificity = (the number of patients with a predicted probability of survival < 10%)/(the number of patients who actually died before the end of observation period);

(3) positive predicted value (PPV) = (the number of patients who actually survive the whole observation period)/(the number of patients with a predicted probability of survival > = 10%);

(4) negative predicted value (NPV) = (the number of patients who actually died before the end of observation period)/(the number of patients with a predicted probability of survival < 10%).

Sensitivity measured the proportion of patients with a correct predicted outcome among those actually surviving the whole observation period. Specificity measured the proportion of patients with a correct predicted outcome among those actually passing away during the observation period. PPV indicated the proportion of patients who did survive the whole observation period among those who were expected to survive. NPV showed the proportion of patients who did die during the observation period among those who were expected to die. As shown by these formulae, the directions of changes in sensitivity and in specificity are reversed, and the directions of changes in PPV and in NPV are reversed.

We emphasized NPV when assessing the quality of predictions, because we aimed to reduce the number of patients being wrongly classified into a group with anticipated death in a near future (called "false negative cases" in statistics). During communication concerning future healthcare for a PMV patient, data on NPV can provide the patient and the family reference information in regard to whether a patient with an anticipated death in a near future according to PMV patients' prognosis in the past would really pass away soon. Using this standard of assessing the quality of predictions, we inevitably had a low accuracy level for detecting death cases among those who actually died.

We also calculated one more performance measure: the c-statistic. This measure is also termed as AUC, which indicates the area under the receiver operating characteristic curve. It assesses the extent to which predicted outcomes discriminate between subjects with different actual outcomes. In addition to using the log-likelihood ratio test to examine model significance, we used the c-statistic to evaluate the overall adequacy of a prediction model.

## Results

### Study participants and their survival rates

**Figure 1:**
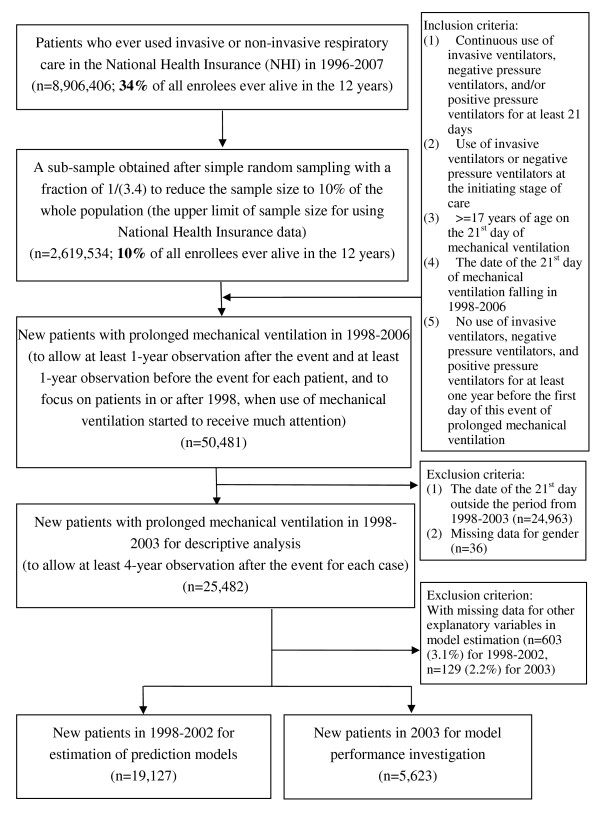
**Flow diagram of the selection process of study subjects**.

There were 25,482 new patients during 1998-2003. In estimation of prediction models and following post-estimation calculations, we further excluded 732 patients with missing data for potential predictors in model estimation. Modelling survival prediction used 19,127 new patients in 1998-2002, and validation of model performance used 5,623 patients of year 2003. Details about the selection process of study subjects are in Figure 1.

Among new PMV patients in 1998-2003, the 3-month survival rate was 51.4%, the 1-year survival rate was 31.9%, and only 17.5% of these patients could survive 4 years (Table 1). Compared to new patients of years in the late 1990s, new patients in years after 2000 generally had higher survival rates in the immediate 3-month period after PMV, but their 3-year and 4-year survival rates appeared poorer (Table 1).

**Table 1:** Post-PMV survival rates, by year of PMV onset (%)

Year Time of follow-up	1998	1999	2000	2001	2002	2003	1998-2003
	*n *= 2,537	*n *= 3,699	*n *= 3,770	*n *= 4,546	*n *= 5,178	*n *= 5,752	*n *= 25,482
3 months	49.7	50.5	49.1	50.8	52.2	54.0	51.4
6 months	40.2	40.3	39.5	39.6	41.5	43.1	40.9
1 year	32.2	31.3	31.0	30.4	32.2	33.7	31.9
2 years	25.8	24.3	24.0	23.3	24.3	25.1	24.4
3 years	23.1	20.3	20.5	20.0	19.5	20.2	20.4
4 years	21.2	17.8	18.3	16.6	16.4	16.7	17.5

*PMV *prolonged mechanical ventilation

### Factors associated with survival

We reported results from the logistic models with the whole set of explanatory variables. Models with alternative specifications had results very similar to this set of models, but generally had lower values for the c-statistic. Log-likelihood ratio test for examining model significance also tended to favour the models reported here. These survival-prediction models identified many factors significantly associated with post-PMV survival (Table 2 and Additional file 2; all ORs were adjusted for all other covariates). Each survival model had a c-statistic > = 0.7, suggesting that the model performance had an acceptable level (see Additional file 3).

**Table 2:** Adjusted odds ratios for selected predictors of post-PMV survival and sample characteristics*

	3-month survival		6-month survival		1-year survival		2-year survival		% patients with the feature
**Predictor**^**‡**^	**OR**^**†**^	95% CI	**OR**^**†**^	95% CI	**OR**^**†**^	95% CI	**OR**^**†**^	95% CI	
**Diagnoses at PMV onset (excluding respiratory failure)**									
Neoplasms	0.30**	0.27-0.34	0.31**	0.27-0.36	0.30**	0.26-0.35	0.31**	0.26-0.38	13.6
Acute and unspecified renal failure	0.39**	0.34-0.45	0.40**	0.35-0.47	0.48**	0.41-0.57	0.56**	0.47-0.67	7.7
Shock	0.42**	0.38-0.47	0.47**	0.42-0.53	0.55**	0.48-0.62	0.63**	0.55-0.73	11.2
Chronic renal failure	0.44**	0.37-0.51	0.43**	0.36-0.52	0.41**	0.33-0.50	0.43**	0.34-0.54	6.3
Septicemia	0.52**	0.48-0.57	0.56**	0.51-0.61	0.60**	0.54-0.66	0.63**	0.56-0.70	20.3
Non-alcoholic liver disease	0.60**	0.51-0.71	0.67**	0.56-0.81	0.72**	0.60-0.88	0.84	0.69-1.03	4.5
Heart failure	0.77**	0.68-0.87	0.76**	0.67-0.86	0.79**	0.69-0.91	0.77**	0.66-0.90	9.1
Diabetes mellitus	0.78**	0.71-0.85	0.77**	0.70-0.85	0.72**	0.65-0.79	0.73**	0.65-0.81	21.0
**Disease history (number of admissions for the disease in the previous year; excluding respiratory failure)**									
Other diseases of the blood and blood-forming organs	0.85**	0.80-0.91	0.83**	0.77-0.90	0.78**	0.72-0.86	0.80**	0.73-0.89	12.6
Non-alcoholic liver disease	0.86**	0.80-0.93	0.86**	0.79-0.94	0.89*	0.81-0.98	0.92	0.83-1.01	6.8
Neoplasms	0.91**	0.88-0.95	0.89**	0.84-0.93	0.88**	0.83-0.93	0.89**	0.84-0.94	14.4
Chronic renal failure	0.91**	0.85-0.97	0.88**	0.81-0.95	0.83**	0.75-0.92	0.78**	0.69-0.89	6.9
**Gender (ref: female)**									
Male	0.89**	0.83-0.95	0.84**	0.78-0.90	0.79**	0.74-0.85	0.78**	0.72-0.84	60.1
**Age group (ref: 17-34)**									
35-44	0.67**	0.51-0.87	0.64**	0.49-0.83	0.61**	0.47-0.79	0.65**	0.51-0.84	3.3
45-54	0.67**	0.52-0.85	0.60**	0.48-0.76	0.54**	0.43-0.69	0.55**	0.44-0.70	6.2
55-64	0.62**	0.49-0.78	0.52**	0.41-0.65	0.43**	0.35-0.54	0.39**	0.32-0.49	11.2
65-74	0.44**	0.36-0.55	0.36**	0.29-0.44	0.28**	0.23-0.35	0.25**	0.20-0.31	26.7
75-84	0.36**	0.29-0.44	0.27**	0.21-0.33	0.19**	0.15-0.23	0.16**	0.13-0.20	35.2
≥ 85	0.29**	0.23-0.36	0.21**	0.17-0.26	0.15**	0.12-0.19	0.11**	0.09-0.14	14.7
Number of patients		19,127*		19,127*		19,127*		19,127*	25,482*
Model significance (results from log-likelihood ratio tests)	χ^2^(103) = 3,099.81**		χ^2^(103) = 3,032.36**		χ^2^(103) = 2,939.46**		χ^2^(103) = 2,652.06**		

*PMV *prolonged MV

*OR *odds-ratio.

*CI *confidence interval.

* *p *< 0.05; ** *p *< 0.01.

* The sample for survival model estimation includes patients from 1998 to 2002, and the sample for describing patient characteristics includes patients from 1998 to 2003.

^† ^The OR was adjusted for all other covariates. Covariates in the model included hospital characteristics at the episode onset (hospital type, hospital location), and individual demographics (gender, age, NHI registration location), year of incidence, socioeconomic conditions (salary class in NHI registration), diseases reported at the episode onset (except respiratory failure), and diseases causing hospital care use within the year before the episode (except respiratory failure).

^‡ ^The table only shows OR figures for selected explanatory variables; OR figures for all explanatory variables are shown in a supplementary file (Additional file 2).

Five disease types are particularly noteworthy: neoplasm, acute and unspecific renal failure, shock, chronic renal failure and septicemia (Table 2). Each had a substantial prevalence rate, and was associated with a low OR that indicated significantly lower survival (OR < 0.65, *p *< 0.01). Non-alcoholic liver disease was also a significant problem, with a substantial prevalence rate and a significant associated with lower 3-month and 6-month survival (OR < 0.7, *p *< 0.01). Heart failure and diabetes mellitus were two more salient health conditions associated with lower survival (OR < 0.8, *p *< 0.01). (See Additional file 2 for OR figures for all other diseases.)

Several diseases that caused hospital care use within one year before PMV were also associated with post-PMV survival. Four disease categories especially call for further research: neoplasm, other diseases of blood and blood-forming organs (the ICD-9 code = 289), chronic renal failure, and non-alcoholic liver disease (Table 2). The numbers of admissions for treating these diseases in the immediate 1-year period before PMV were significantly associated with lower post-PMV survival.

An older age was significantly associated with worse survival, and males had poorer survival (Table 2). With all other factors being controlled for, new patients of a later year tended to have higher survival, suggesting an increasing time trend in adjusted survival (Additional file 2). Patients' demographics and socioeconomic conditions, as well as hospital characteristics at the PMV onset, were related to survival (Additional file 2). In general, patients in the middle tertile of NHI salary class seemed to have poorer survival than those in the other two tertiles. Patients in the very south region in Taiwan (the Kao-Ping region) appeared to have poorer survival, and those staying in a medical centre at the PMV onset tended to have higher survival.

### Performance of survival models

With 10% being the cutoff value for predicated probability, 78 (1.4%), 238, (4.2%), 604 (10.7%) and 1,193 (21.2%) patients among those becoming under PMV in 2003 were chosen as patients with anticipated death (negative cases) within 3 months, 6 months, 1 year, and 2 years after PMV, respectively (Table 3). Although only a low to moderate proportion of patients who actually passed away within the observation period were identified by the prediction, the majority of selected patients had corrected predictions, as shown by the NPVs. For 3-month, 6-month, 1-year and 2-year prediction models, the NPVs were, respectively, 87.2%, 87.0%, 89.9%, and 92.1% (Table 3).

**Table 3:** Performance measures of survival models, with the cutoff value for predicted probability = 10%,* for the 2003 sample

Measure	**Sensitivity**^**†**^			**Specificity**^**†**^			**PPV**^**†**^			**NPV**^**†**^		
	n	%	(se)	n	%	(se)	n	%	(se)	n	%	(se)
3-month survival	2974/2984	99.7	(0.1)	68/2639	2.6	(0.3)	2974/5545	53.6	(0.7)	68/78	87.2	(3.8)
6-month survival	2329/2360	98.7	(0.2)	207/3263	6.3	(0.4)	2329/5385	43.3	(0.7)	207/238	87.0	(2.2)
1-year survival	1762/1823	96.7	(0.4)	543/3800	14.3	(0.6)	1762/5019	35.1	(0.7)	543/604	89.9	(1.2)
2-year survival	1235/1329	92.9	(0.7)	1099/4294	25.6	(0.7)	1235/4430	27.9	(0.7)	1099/1193	92.1	(0.8)

PPV: positive predictive value

NPV: negative predictive value

se: standard error

* As 10% was chosen as the cutoff value for predicted probability, patients with a predicted probability of survival < 10% were classified as a group that would die before the end of observation period, and those with a predicted probability of survival > = 10% were classified as a group that would survive the whole observation period. By comparison of predicted outcomes with actual outcomes, we further calculated the four measures for reflecting the quality of predictions in different perspectives.

^† ^The formulae for these measures are below:

sensitivity = (the number of patients with a predicted probability of survival > = 10%)/(the number of patients who actually survive the whole observation period);

specificity = (the number of patients with a predicted probability of survival < 10%)/(the number of patients who actually died before the end of observation period);

PPV = (the number of patients who actually survive the whole observation period)/(the number of patients with a predicted probability of survival > = 10%);

NPV = (the number of patients who actually died before the end of observation period)/(the number of patients with a predicted probability of survival < 10%).

One more measure reflecting the quality of predictions is the proportion of subjects with corrected predictions. With the 10% cutoff value, 54.1%, 45.1%, 41.0%, and 41.5% among all patients of year 2003 had correct predictions in the 3-month, 6-month, 1-year and 2-year prediction models (Additional file 4). The low rate of correct classification was caused by our conservative threshold for identifying patients with anticipated death. With 50% being the cutoff value, we would have a much higher rate of correct classification. Nevertheless, sensitivity suffers in this case (see Additional file 4). At this threshold, 65.6%, 66.8%, 71.4%, and 78.6% among all patients of year 2003 had correct predictions in the 3-month, 6-month, 1-year and 2-year prediction models (Additional file 4).

### The number of days alive free of hospital stays requiring MV services following a PMV incidence

Inpatient NHI data for new PMV patients of year 2003 who had a predicted survival probability < 10% suggest that they would spend most of their rest of life in hospital stays requiring MV services (Table 4). During the 4-year follow-up time, 75% among patients with anticipated death within 6 months after PMV (the 6-month survival rate < 10%) had 15 or fewer days free of hospital stays requiring MV, and 75% among patients with anticipated death within 2 years after PMV (the 2-year survival rate < 10%) had 25 or fewer days free of hospital stays requiring MV. In contrast, for the whole sample of 2003 PMV patients, the length of time free of ventilation-related inpatient services during the 4-year follow-up time was significantly longer; at least 25% of the patients could have 175 or more days free of hospital stays requiring MV. In the 4-year follow-up period, the mean number of days free of hospital stays requiring MV was 66.0 in those with a predicted 6-month survival rate < 10%, and 111.3 in those with a predicted 2-year survival rate < 10%. In the whole sample of patients in 2003, the mean number of days was 256.9.

**Table 4:** Number of days free of hospital care related to MV in the 4-year after PMV*

	**min**.	25%	Median	75%	80%	85%	90%	95%	**max**.	mean	SD
**Cases with a predicted probability for 3-month survival < 10%**^†^ **(n = 78, 1.4% of the 2003 sample; 87.2% of them were actually deceased within 3 months after PMV.)**											
Number of days free of hospital stays requiring MV	0	0	3	15	19	27	38	279	1,331	39.8	165.2
**Cases with a predicted probability for 6-month survival < 10%**^†^ **(n = 238, 4.2% of the 2003 sample; 87.0% of them were actually deceased within 6 months after PMV.)**											
Number of days free of hospital stays requiring MV	0	0	1	13	17	34	111	385	1,459	66.0	242.3
**Cases with a predicted probability for 1-year survival < 10%**^†^ **(n = 604, 10.7% of the 2003 sample; 89.9% of them were actually deceased within 1 year after PMV.)**											
Number of days free of hospital stays requiring MV	0	0	1	17	31	68	174	570	1,460	90.2	285.8
**Cases with a predicted probability for 2-year survival < 10%**^†^ **(n = 1,193, 21.2% of the 2003 sample; 92.1% of them were actually deceased within 2 years after PMV.)**											
Number of days free of hospital stays requiring MV	0	0	2	24	43	95	280	1,018	1,460	111.3	318.1
**The whole sample of year 2003 (n = 5,623)**											
Number of days free of hospital stays requiring MV	0	0	6	175	382	834	1,383	1,447	1,460	256.9	482.2

*PMV *prolonged MV

*SD *standard deviation

* For this description, we used data for the 2003 patient sample, which was the external validation sample for the survival prediction models.

^† ^The predicted outcome for these patients was death.

## Discussion

Among these PMV patients, the 3-month survival rate was 51.4%, the 1-year survival rate was 31.9%, and only 17.5% of them could survive 4 years. Common health conditions with significant associations with poor survival included neoplasm, acute and unspecific renal failure, chronic renal failure, non-alcoholic liver disease, shock and septicaemia. During a 4-year follow-up period for patients of year 2003, 75% of those with a predicted 6-month survival rate < 10% and 75% of those with a predicted 2-year survival rate < 10% had, respectively, at most 15 and 25 days free of hospital stays requiring MV. In contrast, at least 25% of all patients could have 175 or more days free of hospital stays requiring MV.

### Strengths and limitations

The major strengths of this study include use of a large and nationally representative sample of PMV patients, and the long follow-up time. The rich content and reliable quality of NHI data and death certificate data add additional strengths to this study. Our investigation of "the total length of time free of hospital stays requiring MV in following years after a PMV onset" increases knowledge on post-PMV prognosis, as past studies mainly examined survival rates and in-hospital weaning rates. This study also identifies several common health conditions with significant associations with poor survival. Empirical findings from this study can certainly improve reference data for facilitating communication among participants in care planning for PMV patients.

The major limitation for this study is lack of more detailed physiological and biochemical data. Certain biochemical markers and conditions of organ failure are expected to greatly improve the quality of survival predictions. However, the Taiwan government has not incorporated a system of storing such data into the NHI database yet. With insufficient predictors, prediction models in this study can only have fair performance. As the survival prediction models do not have a very strong capability of discriminating between actual survival and death cases, and we have to be conservative in selecting a cutoff value for predicted outcomes, this prediction system can only identify a small proportion of patients who would actually decease soon. This prediction system also lacks capability of providing comprehensive information on patients' cognitive ability, pain and functional status after a PMV onset.

### PMV incidence rates

Taiwanese experience indicates that the incidence rate of PMV is high under a national health insurance program with generous coverage of inpatient MV services. More than 0.1% of all Taiwanese receive PMV annually. This rate appears slightly higher than that for the United States, where around 300,000 patients use this type of healthcare in ICUs, long-term acute care facilities or specialised weaning units per year [13]. In contrast, the rate for a U.K. region with no dedicated weaning unit may be much lower, as a research in this region reports that there were around 70 cases with PMV annually in this region of 900,000 residents in 2002-2006 [14].

### Post-PMV prognosis

We adopted survival, particularly long-term survival, as the main outcome for our investigation, instead of another important performance indicator for MV services: weaning. While the literature presents different definitions for successful weaning [1,15-19], follow-up time for weaning is usually a period of a few days, and an experience of successful weaning is not necessarily followed by long-term liberation from MV or survival. We looked into long-term survival rather than in-hospital survival, the outcome measure in most past studies [1,18,20-29]. To provide information on whether a patient can have anticipant long-term liberation from MV, we used "the length of subsequent time free of hospital stays requiring MV after a PMV onset" as an indicator.

Our study found 3-month and 1-year survival rates (51.4% and 31.9%, respectively) similar to those observed in a U.S. university-based tertiary-care hospital [30], suggesting that patients with PMV have poor survival even in societies with high levels of providing MV care. Persistent poor functional status after a PMV incidence was observed among most patients in a U.S. study that was based on 5 intensive care units [13]. Similarly, our findings suggest that many Taiwanese patients would have poor ability to perform daily activities for most of their life after a PMV incidence. Research has indicated that almost 75% of Taiwanese patients under PMV in hospitals showed suffering from pain or discomfort, and over 60% had poor cognition [31]. Furthermore, even among patients with fair to good cognition during hospital stays with PMV, over 80% were confined to bed, and unable to have any self-care or usual activities [31]. A condition of staying in a hospital for using MV care appears to be significantly associated with poor ability for performing daily life activities. As our study found that many PMV patients in Taiwan tend to spend most of the rest of life staying in hospitals for using MV services, it suggests that persistent low ability for performing daily activities is expected after a PMV incidence.

### Care planning for PMV patients

Over-optimistic expectations for outcomes of PMV may induce overuse of MV care [32]. While MV services may prolong a patient's life, they do not necessarily improve welfare for the patient or the patient's family. Insufficient communication between physicians and patients or their surrogates is a problem potentially causing inappropriate decisions on using long-term MV care [32]. Over recent years, the "paternalistic model" in the health care system has gradually been replaced by a shared decision-making model [33]. How to clearly present evidence on anticipant poor outcomes to facilitate communication for reducing inappropriate use of MV services is a challenge to both physicians and policy makers. For patients with substantial likelihood of being weaned from MV, the healthcare system has to provide necessary care and an ideal care path to facilitate the process of weaning. On the other hand, it is also important that the healthcare system has to provide a mechanism to help those wishing to terminate PMV services after endeavour for survival.

In 2000, the Taiwan government revised the law to allow withholding of life-sustaining treatment under the condition that this is the preference of a patient approaching the end of life or the patient's family. Nevertheless, this revision of law did not make withdrawal of life-sustaining treatment a lawful act. In 2002, the government revised the law again to allow withdrawal of life-sustaining treatment for a patient approaching the end of life who has written a document indicating the preference. Physicians' dilemma in responding to the preference of PMV patients or their families for withdrawing life-sustaining treatment remained for almost a decade after this law revision. Many PMV patients did not write any documents showing their preference for withdrawing MV services before they became permanently unconscious. In such a case, physicians could not terminate a patient's MV, even when they knew that this was the patient's wish or what the patients' family hoped.

In January of 2011, the government further revised the law to conditionally allow terminal withdrawal of MV for permanently unconscious PMV patients approaching the end of life who previously expressed preference for ending life-sustaining treatment but have left no written documents indicating the preference. For such a patient, the family can make a request for terminating PMV services if each of the living spouse, living adult children, living adult grandchildren, and living parents agrees upon the request. The request can only be approved after an ethics committee organized by the patient's hospital evaluates and agrees upon the request. By the law, at least one third of members in such a committee have to be experts in ethics or law, or persons from the public.

Once a family makes such a request, physicians have to communicate with the family, and help the family to submit the request to an ethics committee if appropriate. In some cases, patients' families may seek help from physicians for assessing whether it is appropriate to continue PMV care. The medical society of Taiwan is currently searching for good guidelines in order to well implement such intervention. One of many essential tasks for constructing guidelines is to generate good reference information on disease prognosis that can facilitate assessment of requests to terminate PMV services, communication with families making the requests, and preparation of application materials for the ethical reviews of the requests.

When confronting requests to help with application for termination of PMV services, physicians can first use their patients' data from medical charts and NHI records, and refer to these results shown by NHI data for a national representative sample of PMV patients, to assess the appropriateness of the application. Our findings show that patients who receive PMV and have low 3-month, 6-month, 1-year or 2-year survival likelihood by prediction are highly likely to have very poor outcomes in following years. For appropriate cases, information generated by this study can also serve as reference materials to ethics committees.

### Information system for improving PMV care

To identify patients highly likely to have poor future outcomes is a task of high priority for improving care patterns for PMV patients [34]. This study is an effort to use national health insurance claims and registration data, and death certificate data to construct a prediction model of long-term outcomes of PMV. It aims to advance understanding in this regard, and to generate an information system that can promote reasonable decisions on whether to continue PMV care or to choose another care pattern.

In Taiwan, each NHI enrolee has an electronic identification card. A healthcare institute can retrieve a patient's background information with the patient's card and permission from the government. Thus, it is feasible to establish the information system that we propose at a low cost. The government can use recently updated population data to generate new parameters for prediction every year to help physicians have better communication with other participants involved in clinical decisions in regard to PMV care. Although insurance claims data do not provide detailed information on physiologic and biochemical variables, the discrimination model established by this study has acceptable performance. With additional effort to input important physiologic and biochemical variables into the NHI database for severely ill patients, we may further construct a discrimination model with better performance. Countries with administrative data of public health services may also take advantage of such data to construct an information system for improving clinical communication and decision-making processes with respect to PMV care.

It is certain that even such large amounts of quantitative reference data alone are unable to provide sufficient information for making decisions on healthcare for PMV patients. Nevertheless, better reference data may help patients, their families, physicians and participants in reviewing requests to terminate PMV care make better decisions that advance quality of life for individual patients at the end of life. The endeavour to construct more data on disease prognosis among PMV patients conforms to widely-accepted moral principles for formulating guidelines for clinic and public health practice in a publicly funded healthcare system: (1) respect for autonomy, (2) non-maleficence, (3) beneficence, and (4) justice [35]. Such effort is expected to enhance autonomy in choice of healthcare, and advance more rational discussion on PMV care use.

The final point to be mentioned is that we need to quickly establish more and clearer guidelines on providing MV services to patients with the following diseases: neoplasm, acute and unspecific renal failure, chronic renal failure, and non-alcoholic liver disease. It calls for more research on appropriateness of providing MV to patients at different prognosis stages of these diseases. With more reference information generated from data of large representative samples, we will have better opportunities to construct clinical guidelines with high persuasion and practical value to the public.

## Conclusions

Neoplasm, acute and unspecific renal failure, shock, chronic renal failure, septicemia, and non-alcoholic liver disease are significantly associated with poor survival among PMV patients. Patients with anticipated death in a near future tend to spend most of the rest of their life staying in hospital using MV services. This calls for further research into assessing PMV care need among patients at different prognosis stages of diseases listed above.

## Competing interests

The authors declare that they have no competing interests.

## Authors' contributions

All the authors helped in obtaining research data and funding, and participated in study design and preparation of the draft. HL took major responsibility for data analysis and interpretation. LC oversaw the study and took major responsibility for writing the draft. All authors read and approved the final manuscript.

## Pre-publication history

The pre-publication history for this paper can be accessed here:

http://www.biomedcentral.com/1472-6963/12/100/prepub

## Supplementary Material

Additional file 1 **Disease categorization**. Click here for file

Additional file 2 **Odds ratios for all explanatory variables**. Click here for file

Additional file 3 **Receiver operating characteristic curves**. Click here for file

Additional file 4 **Classification tables for comparing predicted outcomes with actual outcomes**. Click here for file
