# Supplementary material for: Outcomes of prolonged mechanic ventilation: a discrimination model based on longitudinal health insurance and death certificate data
Source: BMC Health Serv Res. 2012 Apr 25;12:100. doi: 10.1186/1472-6963-12-100 (PMC3375202; doi:10.1186/1472-6963-12-100)
Supplement: Additional file 2 — Odds ratios for all explanatory variables. [file 1472-6963-12-100-S2.DOC]

**Adjusted OR figures of all explanatory variables^*^**

|  | **3-month survival** | | | **6-month survival** | | | **1-year survival** | | | **2-year survival** | | | % patients with the  feature |
| --- | --- | --- | --- | --- | --- | --- | --- | --- | --- | --- | --- | --- | --- |
| Predictor | OR^†^ |  | 95% CI | OR^†^ |  | 95% CI | OR^†^ |  | 95% CI | OR^†^ |  | 95% CI |
| **Diagnoses at PMV onset (excluding respiratory failure)** |  |  |  |  |  |  |  |  |  |  |  |  |  |
| Septicemia | 0.52 | ** | 0.48-0.57 | 0.56 | ** | 0.51-0.61 | 0.60 | ** | 0.54-0.66 | 0.63 | ** | 0.56-0.70 | 20.3 |
| Other infectious and parasitic diseases | 0.79 | ** | 0.71-0.88 | 0.81 | ** | 0.72-0.91 | 0.80 | ** | 0.71-0.90 | 0.82 | ** | 0.72-0.94 | 9.9 |
| Neoplasms | 0.30 | ** | 0.27-0.34 | 0.31 | ** | 0.27-0.36 | 0.30 | ** | 0.26-0.35 | 0.31 | ** | 0.26-0.38 | 13.6 |
| Diabetes mellitus | 0.78 | ** | 0.71-0.85 | 0.77 | ** | 0.70-0.85 | 0.72 | ** | 0.65-0.79 | 0.73 | ** | 0.65-0.81 | 21.0 |
| Nutritional deficiencies | 0.74 | * | 0.58-0.93 | 0.88 |  | 0.69-1.13 | 0.84 |  | 0.64-1.10 | 0.71 | * | 0.52-0.99 | 1.7 |
| Fluid and electrolyte disorders | 0.99 |  | 0.83-1.18 | 0.94 |  | 0.78-1.12 | 0.97 |  | 0.81-1.18 | 0.96 |  | 0.78-1.19 | 3.4 |
| Other endocrine/nutritional/metabolic diseases & immunity disorders | 0.83 | ** | 0.72-0.96 | 0.83 | * | 0.71-0.96 | 0.79 | ** | 0.67-0.93 | 0.72 | ** | 0.60-0.86 | 5.3 |
| Coagulation and hemorrhagic disorders | 0.40 | ** | 0.28-0.57 | 0.55 | ** | 0.38-0.80 | 0.61 | * | 0.41-0.92 | 0.74 |  | 0.48-1.14 | 1.1 |
| Other diseases of the blood and blood-forming organs | 1.02 |  | 0.88-1.18 | 1.09 |  | 0.93-1.26 | 0.98 |  | 0.83-1.15 | 0.95 |  | 0.79-1.14 | 5.0 |
| Mental illness | 1.27 | * | 1.02-1.58 | 1.26 | * | 1.01-1.56 | 1.20 |  | 0.96-1.51 | 1.19 |  | 0.94-1.52 | 2.1 |
| Diseases of the nervous system and sense organs | 1.07 |  | 0.96-1.19 | 1.06 |  | 0.95-1.18 | 1.04 |  | 0.93-1.16 | 1.07 |  | 0.95-1.21 | 10.3 |
| Hypertension | 1.32 | ** | 1.19-1.47 | 1.30 | ** | 1.18-1.44 | 1.41 | ** | 1.26-1.56 | 1.39 | ** | 1.24-1.55 | 11.8 |
| Heart valve disorders | 0.92 |  | 0.76-1.10 | 1.02 |  | 0.85-1.23 | 1.18 |  | 0.97-1.43 | 1.17 |  | 0.95-1.45 | 3.4 |
| Acute myocardial infarction and coronary atherosclerosis | 0.88 |  | 0.78-1.00 | 0.98 |  | 0.87-1.12 | 1.10 |  | 0.96-1.26 | 1.23 | ** | 1.06-1.42 | 7.6 |
| Cardiac dysrhythmias | 0.89 |  | 0.77-1.02 | 0.87 |  | 0.75-1.00 | 0.91 |  | 0.78-1.06 | 0.97 |  | 0.82-1.15 | 5.8 |
| Other diseases of the heart | 0.70 | ** | 0.57-0.85 | 0.73 | ** | 0.60-0.90 | 0.76 | * | 0.61-0.95 | 0.82 |  | 0.65-1.05 | 2.5 |
| Cerebrovascular diseases | 1.06 |  | 0.97-1.16 | 1.03 |  | 0.94-1.13 | 1.00 |  | 0.91-1.10 | 0.99 |  | 0.89-1.10 | 20.0 |
| Other diseases of the circulatory system | 0.60 | ** | 0.50-0.72 | 0.73 | ** | 0.60-0.89 | 0.80 | * | 0.65-0.98 | 0.94 |  | 0.75-1.16 | 3.3 |
| Pneumonia | 0.81 | ** | 0.76-0.87 | 0.84 | ** | 0.78-0.90 | 0.86 | ** | 0.80-0.93 | 0.88 | ** | 0.81-0.95 | 42.7 |
| Chronic obstructive pulmonary disease and bronchiectasis | 1.14 | ** | 1.04-1.26 | 1.16 | ** | 1.05-1.28 | 1.10 |  | 0.99-1.23 | 1.10 |  | 0.97-1.24 | 15.1 |
| Asthma | 0.88 |  | 0.73-1.05 | 0.85 |  | 0.70-1.02 | 0.95 |  | 0.78-1.16 | 1.00 |  | 0.80-1.25 | 3.3 |
| Other diseases of the respiratory system | 0.83 | ** | 0.76-0.90 | 0.87 | ** | 0.80-0.95 | 0.89 | * | 0.81-0.97 | 0.92 |  | 0.83-1.01 | 19.3 |
| Gastrointestinal hemorrhage | 0.72 | ** | 0.62-0.83 | 0.74 | ** | 0.63-0.86 | 0.76 | ** | 0.64-0.90 | 0.79 | * | 0.65-0.95 | 5.1 |
| Other diseases of the digestive system | 0.84 | ** | 0.77-0.92 | 0.90 | * | 0.82-0.98 | 0.99 |  | 0.89-1.09 | 0.99 |  | 0.89-1.10 | 16.3 |
| Acute and unspecified renal failure | 0.39 | ** | 0.34-0.45 | 0.40 | ** | 0.35-0.47 | 0.48 | ** | 0.41-0.57 | 0.56 | ** | 0.47-0.67 | 7.7 |
| Urinary tract infections | 1.25 | ** | 1.14-1.36 | 1.19 | ** | 1.09-1.31 | 1.14 | ** | 1.04-1.26 | 1.12 | * | 1.01-1.25 | 16.4 |
| Other diseases of the genitourinary system | 0.78 | ** | 0.69-0.89 | 0.79 | ** | 0.68-0.91 | 0.75 | ** | 0.65-0.88 | 0.78 | ** | 0.66-0.93 | 5.9 |
| Complications: pregnancy/childbirth/puerperium | 0.80 |  | 0.27-2.41 | 0.70 |  | 0.23-2.12 | 0.66 |  | 0.22-2.02 | 0.54 |  | 0.18-1.66 | 0.1 |
| Diseases of the skin and subcutaneous tissue | 0.82 | ** | 0.72-0.94 | 0.75 | ** | 0.65-0.86 | 0.75 | ** | 0.64-0.88 | 0.77 | ** | 0.64-0.92 | 6.2 |
| Diseases of the musculoskeletal system and connective tissue | 0.93 |  | 0.77-1.13 | 1.06 |  | 0.87-1.29 | 1.13 |  | 0.92-1.39 | 1.17 |  | 0.94-1.45 | 2.9 |
| Congenital anomalies | 0.99 |  | 0.59-1.66 | 1.11 |  | 0.67-1.85 | 1.26 |  | 0.75-2.11 | 1.30 |  | 0.77-2.19 | 0.4 |
| Spinal cord injury | 2.00 | ** | 1.30-3.08 | 1.90 | ** | 1.29-2.79 | 1.72 | ** | 1.19-2.49 | 1.74 | ** | 1.21-2.51 | 0.9 |
| Intracranial injury | 1.16 |  | 0.98-1.37 | 1.32 | ** | 1.12-1.56 | 1.30 | ** | 1.10-1.53 | 1.34 | ** | 1.13-1.59 | 4.9 |
| Other injury and poisoning | 0.99 |  | 0.88-1.12 | 1.04 |  | 0.93-1.17 | 1.14 | * | 1.01-1.29 | 1.30 | ** | 1.14-1.47 | 9.9 |
| Symptoms; signs; and ill-defined conditions and factors | 0.89 |  | 0.74-1.07 | 0.87 |  | 0.72-1.06 | 0.82 |  | 0.66-1.01 | 0.91 |  | 0.73-1.14 | 3.0 |
| Shock | 0.42 | ** | 0.38-0.47 | 0.47 | ** | 0.42-0.53 | 0.55 | ** | 0.48-0.62 | 0.63 | ** | 0.55-0.73 | 11.2 |
| Parkinson's disease | 1.80 | ** | 1.26-2.56 | 1.94 | ** | 1.38-2.73 | 1.82 | ** | 1.29-2.57 | 1.32 |  | 0.90-1.93 | 1.0 |
| Heart failure | 0.77 | ** | 0.68-0.87 | 0.76 | ** | 0.67-0.86 | 0.79 | ** | 0.69-0.91 | 0.77 | ** | 0.66-0.90 | 9.1 |
| Alcoholic liver disease | 1.18 |  | 0.70-1.99 | 0.83 |  | 0.49-1.41 | 0.83 |  | 0.48-1.43 | 0.81 |  | 0.45-1.45 | 0.4 |
| Non-alcoholic liver disease | 0.60 | ** | 0.51-0.71 | 0.67 | ** | 0.56-0.81 | 0.72 | ** | 0.60-0.88 | 0.84 |  | 0.69-1.03 | 4.5 |
| Multiple sclerosis/Other hereditary & degenerative nervous system conditions | 0.98 |  | 0.81-1.17 | 1.10 |  | 0.92-1.31 | 1.11 |  | 0.93-1.33 | 1.12 |  | 0.93-1.35 | 3.7 |
| Chronic renal failure | 0.44 | ** | 0.37-0.51 | 0.43 | ** | 0.36-0.52 | 0.41 | ** | 0.33-0.50 | 0.43 | ** | 0.34-0.54 | 6.3 |
| **Disease history (Number of admissions for the disease in the previous year; excluding respiratory failure)** | | | | | | | | | | | | | |
| Septicemia | 1.14 | ** | 1.05-1.24 | 1.12 | ** | 1.03-1.21 | 1.08 |  | 0.98-1.18 | 1.05 |  | 0.94-1.16 | 13.6 |
| Other infectious and parasitic diseases | 0.93 | ** | 0.89-0.98 | 0.95 |  | 0.90-1.00 | 0.96 |  | 0.90-1.01 | 0.94 |  | 0.88-1.00 | 16.8 |
| Neoplasms | 0.91 | ** | 0.88-0.95 | 0.89 | ** | 0.84-0.93 | 0.88 | ** | 0.83-0.93 | 0.89 | ** | 0.84-0.94 | 14.4 |
| Diabetes mellitus | 0.98 |  | 0.94-1.01 | 0.94 | ** | 0.91-0.98 | 0.93 | ** | 0.89-0.97 | 0.91 | ** | 0.87-0.96 | 23.6 |
| Nutritional deficiencies | 0.99 |  | 0.87-1.12 | 0.92 |  | 0.80-1.06 | 0.85 |  | 0.72-1.00 | 0.80 | * | 0.65-0.98 | 3.3 |
| Fluid and electrolyte disorders | 0.96 |  | 0.89-1.04 | 0.96 |  | 0.89-1.05 | 1.06 |  | 0.97-1.16 | 1.05 |  | 0.95-1.16 | 10.2 |
| Other endocrine/nutritional/metabolic diseases & immunity disorders | 1.03 |  | 0.96-1.10 | 1.00 |  | 0.93-1.07 | 1.02 |  | 0.95-1.10 | 1.08 |  | 0.99-1.17 | 12.1 |
| Coagulation and hemorrhagic disorders | 1.07 |  | 0.80-1.43 | 0.91 |  | 0.66-1.25 | 1.00 |  | 0.71-1.40 | 0.86 |  | 0.58-1.27 | 0.9 |
| Other diseases of the blood and blood-forming organs | 0.85 | ** | 0.80-0.91 | 0.83 | ** | 0.77-0.90 | 0.78 | ** | 0.72-0.86 | 0.80 | ** | 0.73-0.89 | 12.6 |
| Mental illness | 0.99 |  | 0.94-1.05 | 1.00 |  | 0.94-1.06 | 1.00 |  | 0.94-1.06 | 0.99 |  | 0.93-1.06 | 7.7 |
| Diseases of the nervous system and sense organs | 1.03 |  | 0.97-1.08 | 1.03 |  | 0.98-1.08 | 1.05 |  | 0.99-1.11 | 0.99 |  | 0.93-1.06 | 17.0 |
| Hypertension | 1.00 |  | 0.96-1.04 | 1.01 |  | 0.97-1.05 | 1.00 |  | 0.96-1.05 | 0.96 |  | 0.91-1.02 | 28.1 |
| Heart valve disorders | 0.99 |  | 0.91-1.08 | 0.96 |  | 0.87-1.05 | 0.94 |  | 0.85-1.04 | 1.00 |  | 0.89-1.11 | 6.2 |
| Acute myocardial infarction and coronary atherosclerosis | 1.00 |  | 0.94-1.05 | 0.98 |  | 0.93-1.04 | 0.96 |  | 0.90-1.03 | 0.95 |  | 0.88-1.02 | 14.2 |
| Cardiac dysrhythmias | 1.05 |  | 0.98-1.13 | 1.04 |  | 0.97-1.12 | 1.04 |  | 0.96-1.13 | 0.99 |  | 0.90-1.09 | 10.7 |
| Other diseases of the heart | 1.14 | ** | 1.03-1.26 | 1.09 |  | 0.99-1.21 | 1.03 |  | 0.92-1.14 | 1.02 |  | 0.91-1.15 | 4.7 |
| Cerebrovascular diseases | 1.11 | ** | 1.06-1.15 | 1.09 | ** | 1.05-1.14 | 1.08 | ** | 1.03-1.12 | 1.06 | * | 1.01-1.11 | 24.5 |
| Other diseases of the circulatory system | 1.04 |  | 0.94-1.16 | 0.99 |  | 0.88-1.11 | 0.99 |  | 0.87-1.12 | 0.98 |  | 0.85-1.12 | 5.7 |
| Pneumonia | 1.14 | ** | 1.09-1.19 | 1.11 | ** | 1.06-1.16 | 1.08 | ** | 1.02-1.13 | 1.04 |  | 0.98-1.10 | 30.9 |
| Chronic obstructive pulmonary disease and bronchiectasis | 1.00 |  | 0.97-1.04 | 1.02 |  | 0.99-1.06 | 1.01 |  | 0.97-1.05 | 1.00 |  | 0.96-1.05 | 20.3 |
| Asthma | 1.04 |  | 0.97-1.11 | 1.04 |  | 0.97-1.11 | 1.07 |  | 0.99-1.15 | 1.01 |  | 0.93-1.10 | 7.0 |
| Other diseases of the respiratory system | 1.03 |  | 0.98-1.08 | 0.99 |  | 0.94-1.04 | 1.00 |  | 0.95-1.05 | 1.03 |  | 0.97-1.09 | 22.6 |
| Gastrointestinal hemorrhage | 1.04 |  | 0.94-1.16 | 0.99 |  | 0.88-1.10 | 0.99 |  | 0.88-1.12 | 1.00 |  | 0.87-1.15 | 6.4 |
| Other diseases of the digestive system | 1.00 |  | 0.96-1.04 | 0.99 |  | 0.95-1.04 | 0.98 |  | 0.93-1.03 | 1.00 |  | 0.95-1.06 | 27.4 |
| Acute and unspecified renal failure | 1.24 | ** | 1.11-1.38 | 1.15 | * | 1.03-1.29 | 0.97 |  | 0.84-1.12 | 0.98 |  | 0.83-1.16 | 5.3 |
| Urinary tract infections | 0.96 |  | 0.92-1.01 | 0.97 |  | 0.93-1.02 | 0.94 | * | 0.89-1.00 | 0.97 |  | 0.91-1.03 | 21.5 |
| Other diseases of the genitourinary system | 0.96 |  | 0.91-1.01 | 0.95 |  | 0.90-1.00 | 0.96 |  | 0.90-1.02 | 0.95 |  | 0.89-1.02 | 18.3 |
| Complications: pregnancy/childbirth/puerperium | 0.82 |  | 0.42-1.62 | 0.92 |  | 0.47-1.82 | 0.84 |  | 0.42-1.65 | 1.06 |  | 0.54-2.10 | 0.1 |
| Diseases of the skin and subcutaneous tissue | 0.94 | * | 0.88-0.99 | 0.92 | ** | 0.86-0.98 | 0.92 | * | 0.86-0.99 | 0.94 |  | 0.87-1.02 | 10.5 |
| Diseases of the musculoskeletal system and connective tissue | 0.97 |  | 0.91-1.04 | 0.94 |  | 0.87-1.01 | 0.92 | * | 0.85-1.00 | 0.92 |  | 0.85-1.01 | 9.2 |
| Congenital anomalies | 0.85 |  | 0.63-1.15 | 0.96 |  | 0.71-1.30 | 1.00 |  | 0.73-1.38 | 1.05 |  | 0.75-1.47 | 0.8 |
| Spinal cord injury | 1.31 |  | 0.99-1.73 | 1.08 |  | 0.86-1.36 | 0.97 |  | 0.78-1.21 | 1.04 |  | 0.83-1.30 | 0.7 |
| Intracranial injury | 1.15 | * | 1.02-1.31 | 1.14 | * | 1.01-1.29 | 1.08 |  | 0.96-1.23 | 1.03 |  | 0.90-1.17 | 4.4 |
| Other injury and poisoning | 0.93 | * | 0.87-0.99 | 0.96 |  | 0.90-1.03 | 0.98 |  | 0.91-1.05 | 0.98 |  | 0.91-1.06 | 14.6 |
| Symptoms; signs; and ill-defined conditions and factors | 0.96 |  | 0.89-1.04 | 0.98 |  | 0.90-1.06 | 0.96 |  | 0.87-1.05 | 0.98 |  | 0.88-1.08 | 10.5 |
| Shock | 1.32 | ** | 1.15-1.52 | 1.25 | ** | 1.08-1.44 | 1.18 | * | 1.01-1.37 | 1.12 |  | 0.94-1.33 | 5.2 |
| Parkinson's disease | 0.98 |  | 0.87-1.09 | 0.95 |  | 0.85-1.07 | 0.99 |  | 0.88-1.11 | 1.01 |  | 0.89-1.16 | 2.4 |
| Heart failure | 0.94 | * | 0.88-1.00 | 0.95 |  | 0.89-1.02 | 0.91 | * | 0.84-0.98 | 0.91 | * | 0.84-1.00 | 11.6 |
| Alcoholic liver disease | 0.94 |  | 0.76-1.18 | 0.92 |  | 0.73-1.17 | 0.86 |  | 0.67-1.12 | 0.72 | * | 0.53-0.97 | 0.8 |
| Non-alcoholic liver disease | 0.86 | ** | 0.80-0.93 | 0.86 | ** | 0.79-0.94 | 0.89 | * | 0.81-0.98 | 0.92 |  | 0.83-1.01 | 6.8 |
| Multiple sclerosis/Other hereditary & degenerative nervous system conditions | 1.11 |  | 0.99-1.25 | 1.08 |  | 0.96-1.21 | 1.02 |  | 0.91-1.15 | 0.97 |  | 0.85-1.11 | 4.0 |
| Chronic renal failure | 0.91 | ** | 0.85-0.97 | 0.88 | ** | 0.81-0.95 | 0.83 | ** | 0.75-0.92 | 0.78 | ** | 0.69-0.89 | 6.9 |
| **Hospital accreditation level (ref: medical centre)** |  |  |  |  |  |  |  |  |  |  |  |  |  |
| Regional hospital | 0.92 | * | 0.86-1.00 | 0.89 | ** | 0.82-0.96 | 0.85 | ** | 0.79-0.93 | 0.87 | ** | 0.80-0.95 | 36.5 |
| Local hospital | 0.90 | * | 0.82-0.98 | 0.86 | ** | 0.79-0.95 | 0.87 | ** | 0.79-0.96 | 0.85 | ** | 0.77-0.95 | 20.3 |
| **Region of hospital location (ref: Taipei)** |  |  |  |  |  |  |  |  |  |  |  |  |  |
| Northern region | 0.89 | * | 0.80-1.00 | 0.94 |  | 0.83-1.05 | 0.99 |  | 0.87-1.12 | 0.99 |  | 0.86-1.13 | 11.5 |
| Central region | 1.13 | ** | 1.03-1.24 | 1.14 | ** | 1.04-1.25 | 1.12 | * | 1.01-1.24 | 1.13 | * | 1.01-1.26 | 24.0 |
| Southern region | 1.04 |  | 0.93-1.17 | 0.97 |  | 0.87-1.09 | 0.87 | * | 0.77-0.99 | 0.86 | * | 0.75-0.99 | 13.1 |
| Kao-Ping region | 0.89 | * | 0.81-0.98 | 0.88 | * | 0.80-0.97 | 0.84 | ** | 0.75-0.93 | 0.82 | ** | 0.73-0.93 | 17.9 |
| Eastern region | 0.92 |  | 0.77-1.10 | 1.00 |  | 0.83-1.20 | 0.87 |  | 0.71-1.06 | 0.90 |  | 0.73-1.12 | 3.6 |
| **PMV incidence year** |  |  |  |  |  |  |  |  |  |  |  |  |  |
| continuous variable in the value range: [1998, 2002] | 1.06 | ** | 1.04-1.09 | 1.06 | ** | 1.04-1.09 | 1.07 | ** | 1.04-1.09 | 1.06 | ** | 1.03-1.09 |  |
| **Gender (ref: female)** |  |  |  |  |  |  |  |  |  |  |  |  |  |
| Male | 0.89 | ** | 0.83-0.95 | 0.84 | ** | 0.78-0.90 | 0.79 | ** | 0.74-0.85 | 0.78 | ** | 0.72-0.84 | 60.1 |
| **Age group (ref: 17–34)** |  |  |  |  |  |  |  |  |  |  |  |  |  |
| 35–44 | 0.67 | ** | 0.51-0.87 | 0.64 | ** | 0.49-0.83 | 0.61 | ** | 0.47-0.79 | 0.65 | ** | 0.51-0.84 | 3.3 |
| 45–54 | 0.67 | ** | 0.52-0.85 | 0.60 | ** | 0.48-0.76 | 0.54 | ** | 0.43-0.69 | 0.55 | ** | 0.44-0.70 | 6.2 |
| 55–64 | 0.62 | ** | 0.49-0.78 | 0.52 | ** | 0.41-0.65 | 0.43 | ** | 0.35-0.54 | 0.39 | ** | 0.32-0.49 | 11.2 |
| 65–74 | 0.44 | ** | 0.36-0.55 | 0.36 | ** | 0.29-0.44 | 0.28 | ** | 0.23-0.35 | 0.25 | ** | 0.20-0.31 | 26.7 |
| 75–84 | 0.36 | ** | 0.29-0.44 | 0.27 | ** | 0.21-0.33 | 0.19 | ** | 0.15-0.23 | 0.16 | ** | 0.13-0.20 | 35.2 |
| ≥85 | 0.29 | ** | 0.23-0.36 | 0.21 | ** | 0.17-0.26 | 0.15 | ** | 0.12-0.19 | 0.11 | ** | 0.09-0.14 | 14.7 |
| **NHI registration location (ref: big city)** |  |  |  |  |  |  |  |  |  |  |  |  |  |
| Small city or town | 1.02 |  | 0.93-1.11 | 1.04 |  | 0.95-1.13 | 1.02 |  | 0.93-1.13 | 1.04 |  | 0.94-1.15 | 40.9 |
| Remote or rural area | 0.95 |  | 0.87-1.04 | 0.98 |  | 0.90-1.07 | 1.02 |  | 0.93-1.13 | 1.04 |  | 0.94-1.15 | 20.6 |
| **Salary class in NHI registration (ref: bottom 1/3 class)** |  |  |  |  |  |  |  |  |  |  |  |  |  |
| The middle 1/3 of the population | 0.85 | ** | 0.79-0.91 | 0.85 | ** | 0.79-0.92 | 0.89 | ** | 0.82-0.97 | 0.88 | ** | 0.81-0.96 | 40.9 |
| The top 1/3 of the population | 1.08 |  | 0.99-1.17 | 1.12 | * | 1.02-1.22 | 1.08 |  | 0.98-1.19 | 1.07 |  | 0.97-1.18 | 20.6 |
| Number of patients |  |  | 19,127* |  |  | 19,127* |  |  | 19,127* |  |  | 19,127* | 25,482* |
| Model significance (results from log-likelihood ratio tests) | χ^2^(103)=3,099.81^**^ | | | χ^2^(103)=3,032.36^**^ | | | χ^2^(103)=2,939.46^**^ | | | χ^2^(103)=2,652.06^**^ | | |  |

PMV: prolonged mechanical ventilation

OR: odds-ratio.

CI: confidence interval.

^*^ *p*<0.05; ^**^ *p*<0.01.

**^*^** The sample for survival model estimation includes patients from 1998 to 2002, and the sample for describing patient characteristics includes patients from 1998 to 2003.

^†^ The OR was adjusted for all other covariates.
