# Supplementary material for: Outcomes of prolonged mechanic ventilation: a discrimination model based on longitudinal health insurance and death certificate data
Source: BMC Health Serv Res. 2012 Apr 25;12:100. doi: 10.1186/1472-6963-12-100 (PMC3375202; doi:10.1186/1472-6963-12-100)
Supplement: Additional file 3 — Receiver operating characteristic curves. [file 1472-6963-12-100-S3.DOC]

**ROC curves for survival models selected as final models in the paper**

| 3-month survival model  (C-statistic=0.7016, se=0.006868, 95% CI: 0.6881-0.7151) | 6-month survival model  (C-statistic=0.7146, se=0.007064, 95% CI: 0.7008-0.7284) |
| --- | --- |
| 1-year survival model  (C-statistic=0.7234, se=0.007582, 95% CI: 0.7085-0.7383) | 2-year survival model  (C-statistic=0.7394, se=0.008421, 95% CI: 0.7229-0.7559) |

ROC: receiver operating characteristic; se: standard error; CI: confidence interval.
