# Supplementary material for: Outcomes of prolonged mechanic ventilation: a discrimination model based on longitudinal health insurance and death certificate data
Source: BMC Health Serv Res. 2012 Apr 25;12:100. doi: 10.1186/1472-6963-12-100 (PMC3375202; doi:10.1186/1472-6963-12-100)
Supplement: Additional file 4 — Classification tables for comparing predicted outcomes with actual outcomes. [file 1472-6963-12-100-S4.DOC]

**Quality of predictions: comparison of predicted outcomes with actual outcomes**

**Part 1: Classification tables for the 3-month survival model**

| (A) The cutoff value for predicted probability is 0.1  -------- True --------  Classified | D ~D | Total  -----------+--------------------------+-----------  + | 2974 2571 | 5545  - | 10 68 | 78  -----------+--------------------------+-----------  Total | 2984 2639 | 5623    Classified + if predicted Pr(D) >= .1  True D defined as alive_3m != 0  --------------------------------------------------  Sensitivity Pr( +| D) 99.66%  Specificity Pr( -|~D) 2.58%  Positive predictive value Pr( D| +) 53.63%  Negative predictive value Pr(~D| -) 87.18%  --------------------------------------------------  False + rate for true ~D Pr( +|~D) 97.42%  False - rate for true D Pr( -| D) 0.34%  False + rate for classified + Pr(~D| +) 46.37%  False - rate for classified - Pr( D| -) 12.82%  --------------------------------------------------  Correctly classified 54.10%  -------------------------------------------------- | (B) The cutoff value for predicted probability is 0.5 (optimal)  -------- True --------  Classified | D ~D | Total  -----------+--------------------------+-----------  + | 2085 1037 | 3122  - | 899 1602 | 2501  -----------+--------------------------+-----------  Total | 2984 2639 | 5623    Classified + if predicted Pr(D) >= .5  True D defined as alive_3m != 0  --------------------------------------------------  Sensitivity Pr( +| D) 69.87%  Specificity Pr( -|~D) 60.70%  Positive predictive value Pr( D| +) 66.78%  Negative predictive value Pr(~D| -) 64.05%  --------------------------------------------------  False + rate for true ~D Pr( +|~D) 39.30%  False - rate for true D Pr( -| D) 30.13%  False + rate for classified + Pr(~D| +) 33.22%  False - rate for classified - Pr( D| -) 35.95%  --------------------------------------------------  Correctly classified 65.57%  -------------------------------------------------- |
| --- | --- |

**Part 2: Classification tables for the 6-month survival model**

| (A) The cutoff value for predicted probability is 0.1  -------- True --------  Classified | D ~D | Total  -----------+--------------------------+-----------  + | 2329 3056 | 5385  - | 31 207 | 238  -----------+--------------------------+-----------  Total | 2360 3263 | 5623    Classified + if predicted Pr(D) >= .1  True D defined as alive_half != 0  --------------------------------------------------  Sensitivity Pr( +| D) 98.69%  Specificity Pr( -|~D) 6.34%  Positive predictive value Pr( D| +) 43.25%  Negative predictive value Pr(~D| -) 86.97%  --------------------------------------------------  False + rate for true ~D Pr( +|~D) 93.66%  False - rate for true D Pr( -| D) 1.31%  False + rate for classified + Pr(~D| +) 56.75%  False - rate for classified - Pr( D| -) 13.03%  --------------------------------------------------  Correctly classified 45.10%  -------------------------------------------------- | (B) The cutoff value for predicted probability is 0.5 (optimal)  -------- True --------  Classified | D ~D | Total  -----------+--------------------------+-----------  + | 1136 643 | 1779  - | 1224 2620 | 3844  -----------+--------------------------+-----------  Total | 2360 3263 | 5623    Classified + if predicted Pr(D) >= .5  True D defined as alive_half != 0  --------------------------------------------------  Sensitivity Pr( +| D) 48.14%  Specificity Pr( -|~D) 80.29%  Positive predictive value Pr( D| +) 63.86%  Negative predictive value Pr(~D| -) 68.16%  --------------------------------------------------  False + rate for true ~D Pr( +|~D) 19.71%  False - rate for true D Pr( -| D) 51.86%  False + rate for classified + Pr(~D| +) 36.14%  False - rate for classified - Pr( D| -) 31.84%  --------------------------------------------------  Correctly classified 66.80%  -------------------------------------------------- |
| --- | --- |

**Part 3: Classification tables for the 1-year survival model**

| (A) The cutoff value for predicted probability is 0.1  -------- True --------  Classified | D ~D | Total  -----------+--------------------------+-----------  + | 1762 3257 | 5019  - | 61 543 | 604  -----------+--------------------------+-----------  Total | 1823 3800 | 5623    Classified + if predicted Pr(D) >= .1  True D defined as alive_1 != 0  --------------------------------------------------  Sensitivity Pr( +| D) 96.65%  Specificity Pr( -|~D) 14.29%  Positive predictive value Pr( D| +) 35.11%  Negative predictive value Pr(~D| -) 89.90%  --------------------------------------------------  False + rate for true ~D Pr( +|~D) 85.71%  False - rate for true D Pr( -| D) 3.35%  False + rate for classified + Pr(~D| +) 64.89%  False - rate for classified - Pr( D| -) 10.10%  --------------------------------------------------  Correctly classified 40.99%  -------------------------------------------------- | (B) The cutoff value for predicted probability is 0.5 (optimal)  -------- True --------  Classified | D ~D | Total  -----------+--------------------------+-----------  + | 548 335 | 883  - | 1275 3465 | 4740  -----------+--------------------------+-----------  Total | 1823 3800 | 5623    Classified + if predicted Pr(D) >= .5  True D defined as alive_1 != 0  --------------------------------------------------  Sensitivity Pr( +| D) 30.06%  Specificity Pr( -|~D) 91.18%  Positive predictive value Pr( D| +) 62.06%  Negative predictive value Pr(~D| -) 73.10%  --------------------------------------------------  False + rate for true ~D Pr( +|~D) 8.82%  False - rate for true D Pr( -| D) 69.94%  False + rate for classified + Pr(~D| +) 37.94%  False - rate for classified - Pr( D| -) 26.90%  --------------------------------------------------  Correctly classified 71.37%  -------------------------------------------------- |
| --- | --- |

**Part 4: Classification tables for the 2-year survival model**

| (A) The cutoff value for predicted probability is 0.1  -------- True --------  Classified | D ~D | Total  -----------+--------------------------+-----------  + | 1235 3195 | 4430  - | 94 1099 | 1193  -----------+--------------------------+-----------  Total | 1329 4294 | 5623    Classified + if predicted Pr(D) >= .1  True D defined as alive_2 != 0  --------------------------------------------------  Sensitivity Pr( +| D) 92.93%  Specificity Pr( -|~D) 25.59%  Positive predictive value Pr( D| +) 27.88%  Negative predictive value Pr(~D| -) 92.12%  --------------------------------------------------  False + rate for true ~D Pr( +|~D) 74.41%  False - rate for true D Pr( -| D) 7.07%  False + rate for classified + Pr(~D| +) 72.12%  False - rate for classified - Pr( D| -) 7.88%  --------------------------------------------------  Correctly classified 41.51%  -------------------------------------------------- | (B) The cutoff value for predicted probability is 0.5 (optimal)  -------- True --------  Classified | D ~D | Total  -----------+--------------------------+-----------  + | 290 167 | 457  - | 1039 4127 | 5166  -----------+--------------------------+-----------  Total | 1329 4294 | 5623    Classified + if predicted Pr(D) >= .5  True D defined as alive_2 != 0  --------------------------------------------------  Sensitivity Pr( +| D) 21.82%  Specificity Pr( -|~D) 96.11%  Positive predictive value Pr( D| +) 63.46%  Negative predictive value Pr(~D| -) 79.89%  --------------------------------------------------  False + rate for true ~D Pr( +|~D) 3.89%  False - rate for true D Pr( -| D) 78.18%  False + rate for classified + Pr(~D| +) 36.54%  False - rate for classified - Pr( D| -) 20.11%  --------------------------------------------------  Correctly classified 78.55%  -------------------------------------------------- |
| --- | --- |
